# Rosuvastatin-Induced Myopathy: A Case Series

**DOI:** 10.7759/cureus.66180

**Published:** 2024-08-05

**Authors:** Susheel K Malani, Sridevi Chigullapalli, Saurabh Sujanyal, Vijay Sharma

**Affiliations:** 1 Cardiology, Dr. D. Y. Patil Medical College, Hospital and Research Centre, Dr. D. Y. Patil Vidyapeeth, Pune (Deemed to Be University), Pune, IND; 2 Medicine and Surgery, Dr. D. Y. Patil Medical College, Hospital and Research Centre, Dr. D. Y. Patil Vidyapeeth, Pune (Deemed to Be University), Pune, IND

**Keywords:** vitamin d deficiency, statin-induced myopathy, acute liver injury, acute kidney injury, rosuvastatin

## Abstract

Statins are one of the most crucial drugs used for the prevention of atherosclerotic coronary artery disease. A wide spectrum of symptoms ranging from myalgia to symptoms of rhabdomyolysis with or without weakness of the upper and lower limbs are indicative of statin-induced rhabdomyolysis or myopathy. The current case series which represents three patients who developed statin-induced myopathy after starting rosuvastatin is one of a few if not the first case series. All three patients had recently started rosuvastatin 40mg once daily post-percutaneous transluminal coronary angioplasty (PTCA) for secondary prevention of atherosclerotic cardiovascular diseases (ASCVDs). Shortly after starting the medication, they were hospitalized due to bilateral lower limb pain and weakness. On further evaluation, they were diagnosed to have rosuvastatin-induced myopathy with acute kidney injury and/or liver injury. In all cases, myopathy, acute renal injury, and liver injury were caused by rosuvastatin, regardless of the presence of a vitamin D deficiency. Despite the documented risk of myopathy and renal toxicity associated with rosuvastatin, the drug remains highly popular worldwide in the modern period. Although all the cases discussed were successfully treated by stopping rosuvastatin and switching it with another class of lipid-lowering agent, it significantly increased morbidity and raised medical expenses. Hence, this case series not only adds to existing safety disputations associated with rosuvastatin but also calls for more pharmacovigilance when recommending this medication.

## Introduction

Among other countries worldwide, India has the highest burden of cardiovascular diseases (CVDs) with an estimated prevalence in India being 54.5 million [1]. Statins are one of the most crucial drugs used for the prevention of atherosclerotic coronary artery disease (CAD). The quoted incidence of statin-induced myopathy is 0.1-0.2% [2]. A wide spectrum of symptoms ranging from myalgia to symptoms of rhabdomyolysis with or without weakness of the upper and lower limbs are indicative of statin-induced rhabdomyolysis or myopathy [3]. A unique condition known as statin-induced immune-mediated necrotizing myopathy (IMNM) may develop and can worsen even after the statin is stopped. It is thought that anti-3-hydroxy-3-methylglutaryl-coenzyme A reductase (anti-HMGCR) autoantibodies, associated with statin exposure in genetically predisposed people, especially those with the class II major histocompatibility complex (MHC) with the allele DRB1*11:01, play a role, even though the precise pathophysiological mechanism is not fully understood [4]. Here, we describe three cases of statin-induced myopathy that occurred after beginning rosuvastatin medication.

## Case presentation

Case 1

A 62-year-old male came with complaints of vomiting and bilateral lower limb swelling, which was gradually progressive in nature. He had no history of chest pain, fever, rash, cough, joint pain, trauma, strenuous exercise, or addiction. His ongoing medications include rosuvastatin 40mg, aspirin 75mg, ticagrelor 90mg (BD), metoprolol 25mg (BD), and linagliptin 2.5mg, which were started 45 days after he underwent percutaneous transluminal coronary angioplasty (PTCA).

On admission, the patient was hemodynamically stable, and his cardiovascular system (CVS), respiratory system (RS), and per abdominal (PA) examination were normal. On neurological evaluation, there was bilateral upper and lower limb weakness (grade 2/5), proximal more than distal, whereas biceps, triceps, knee, and ankle reflexes were 2/5, and plantar reflex was normal. Cranial nerve, sensory, and higher function were normal.

**Figure 1:**
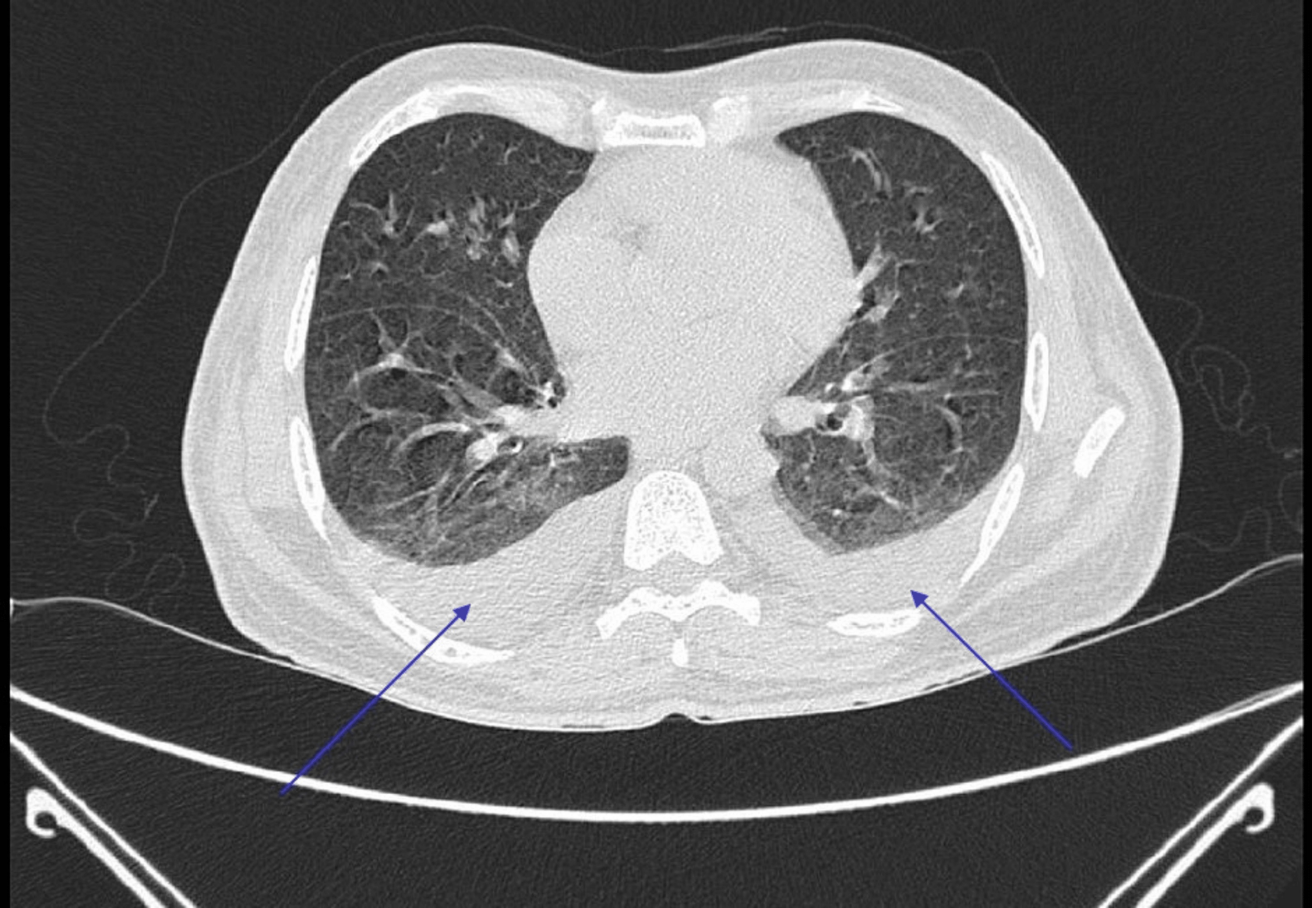
CT scan of the chest showing bilateral pleural effusion (blue arrows)

Laboratory investigation (Table 1) on the day of admission revealed elevated creatine phosphokinase (CPK). Renal function was impaired with elevated creatinine. Arterial blood gas (ABG) analysis was suggestive of high anionic gap metabolic acidosis with elevated lactate levels. Chest computer tomography (CT) scan was suggestive of bilateral pleural effusion (Figure 1). Ultrasonography of the abdomen revealed mild ascites.

**Table 1:** Laboratory investigation on the day of admission for all cases AST: aspartate aminotransferase; ALT: alanine aminotransferase; CPK: creatine phosphokinase; ANA: antinuclear antibody

Parameter	Case 1	Case 2	Case 3	Reference Range
Hemoglobin	10.9 g/dL	11.50 g/dL	8.9 g/dL	11.6-15.0g/dL
Leukocytes	8900 g/µL	5100/µL	12700 /µL	4,000-10,000g/ µL
Platelet	248000/µL	271000/µL	333000/µL	1,50,000-4,10,000/µL
Bilirubin (total)	0.39 mg/dL	1.54 mg/dL	0.36 mg/dL	0.22-1.20 mg/dL
AST	35 U/L	1290 U/L	1074 U/L	8-43U/L
ALT	56 U/L	514 U/L	724 U/L	7-45U/L
Alkaline phosphatase	95 U/L	74 U/L	84 U/L	35-104 U/L
Blood urea nitrogen	102.50 mg/dL	154 mg/dL	212 mg/dL	17-49 mg/dL
Creatinine	6.3 mg/dL	6.26 mg/dL	12.34 mg/dL	0.2-1.2 mg/dL
CPK	2030 U/L	46868 U/L	53240 U/L	<26U/L
Myoglobinuria	Above 3000 µg/L	-	-	28-72 µg/L
Sodium	129.00 mmol/L	133.00 mmol/L	137.00 mmol/L	135-145 mmol/L
Potassium	3.03 mmol/L	3.54 mmol/L	6.32 mmol/L	3.5-5.10 mmol/L
ANA blot	Negative	Negative	Negative	-
Vitamin D	-	-	21	40-100 mg/dL
Calcium	7.80 mg/dL	7.20 mg/dL	7.00 mg/dL	8.5-10.2 mg/dL

Considering these findings, he was diagnosed with rosuvastatin-induced myopathy with acute kidney injury (AKI). The patient was managed initially by stopping rosuvastatin and was started on dialysis. He started showing improvement after five days of stopping rosuvastatin. Rosuvastatin was replaced with bempedoic acid 180mg (OD) to further manage dyslipidemia, and the patient experienced no side effects from this new medication. After stabilization, physiotherapy was initiated to address his lower limb weakness. Renal functions, including serum creatinine levels, gradually normalized after six weeks post-admission. Upper and lower limb power gradually improved with motor recovery at eight weeks.

Case 2

A 70-year-old woman came with complaints of vomiting and generalized body aches. She had a known case of CAD status post-PTCA. In addition to this, she reported an insidious onset of generalized body aches that progressed to the point where she was unable to walk. She had no history of fever, rash, joint pain, chest pain, cough, trauma, strenuous exercise, addiction, or medication allergy. Her current medications included rosuvastatin 40mg, aspirin 75mg, and ticagrelor 90mg (BD), all of which were initiated 75 days after being diagnosed with CAD.

Upon admission, her vitals were stable. Her CVS, RS, and PA examinations were normal. The neurological evaluation suggested bilateral upper and lower limb weakness (grade 3/5), with proximal more than distal muscle weakness, whereas biceps, triceps, knee, and ankle reflexes were 3/5, and plantar reflex was normal. Cranial nerve, sensory, and higher function were normal.

Laboratory investigation (Table 1) on admission revealed elevated CPK and impaired renal function. ABG analysis showed high anionic gap metabolic acidosis with increased lactate levels. These results led to the diagnosis of rosuvastatin-induced myopathy with liver involvement and acute kidney damage (AKI).

Rosuvastatin was discontinued, and AKI was managed conservatively. Improvement was observed after four days of stopping rosuvastatin. To further manage dyslipidemia, rosuvastatin was substituted with bempedoic acid 180mg (OD), to which the patient experienced no side effects. Physiotherapy was initiated for lower limb weakness, and she was discharged on the fifth day with instructions for periodic follow-up. Renal function improved over the next four weeks, and lower limb motor function also improved gradually, with complete recovery at five weeks.

Case 3

A 78-year-old woman was hospitalized due to vomiting and bilateral lower limb progressive weakness over the past 48 hours. She had been taking rosuvastatin 40mg, aspirin 75mg, and ticagrelor 90mg (BD) for the past year after being diagnosed with CAD, which required PTCA.

On admission, she was hemodynamically stable, and CVS, RS, and PA examinations were normal. Neurological evaluation revealed muscle weakness in the lower extremities (grade 2/5), predominantly affecting proximal muscles, whereas biceps, triceps, knee, and ankle reflexes were 3/5, and plantar reflex was normal. Cranial nerve, sensory, and higher functions were normal.

Laboratory investigation (Table 1) on the day of admission revealed elevated CPK. ABG analysis revealed high anionic gap metabolic acidosis with increased lactate levels. Vitamin D levels were found to be in the lower range. An electromyography study was performed on the right iliopsoas, right vastus medialis, right vastus lateralis, and right tibialis anterior showed normal insertion activity with the presence of spontaneous activity as seen in inflammatory myopathy.

Considering these findings, she was concluded to have rosuvastatin-induced myopathy with AKI and liver involvement. Rosuvastatin was withheld, and the patient was started on dialysis. She was started on physiotherapy for lower limb weakness. Bempedoic acid 180mg (OD) was substituted for rosuvastatin to continue managing dyslipidemia, and the patient experienced no side effects from this new medication. Over the next three weeks, her kidney function gradually improved, with creatinine levels normalizing six weeks after admission. Similarly, her lower limb strength improved progressively, with complete motor recovery achieved after six weeks.

## Discussion

Statins rank the most commonly prescribed medications in India for preventing CVD, both in primary and secondary prevention among eligible patients. Statins function by competitively inhibiting the enzyme hydroxymethylglutaryl-CoA (HMG-CoA) reductase, which plays a key role in the conversion of HMG-CoA into mevalonate in the cholesterol synthesis pathway. This inhibition reduces the synthesis of cholesterol in the liver, leading to an increase in the number of low-density lipoprotein (LDL) receptors and enhanced uptake of LDL-cholesterol by the liver from the bloodstream. Common adverse effects of statins include myopathy, hepatotoxicity, and AKI [5].

Four categories of statin-induced myopathy can be distinguished: myositis, self-limited toxic statin myopathy, myalgia or increased creatine kinase, and rhabdomyolysis (i.e. the recently described IMNM with anti-HMGCR antibodies) [6]. Rhabdomyolysis is a most severe form of myopathy characterized by high CK levels (>100 times the upper limit of normality). On the other hand, myalgia or mildly elevated creatine kinase, a mild form of statin-induced muscle toxicity, is characterized by mild CK increase, usually reaching a maximum of up to 1000 IU/L (<5-fold the upper limit of normal). Statin-induced IMNM is a distinct disease that can persist and sometimes progress even after the statin is discontinued. It is thought that anti-HMGCR autoantibodies, associated with statin exposure in genetically predisposed people, especially those with the class II MHC with the allele DRB1*11:01, play a role, even though the precise pathophysiological mechanism is not fully understood [4]. Lastly, self-limited toxic statin myopathy is characterized by myalgia which is predominantly in the proximal muscle, and elevated CK levels, commonly up to 10 and 100 times the upper limit of normal (i.e. 2000-20,000 IU/L) [7].

Rosuvastatin was approved by the Food and Drug Administration (FDA) on July 9, 2003. Nevertheless, soon after its approval, its safety became disputed. Within a few months post-launch, several cases of fulminant rhabdomyolysis were reported in Canada, the United Kingdom, and the United States. Consequently, Public Citizen, a USA (United States of America) non-profit organization, and progressive consumer rights advocacy group, filed a petition to ban rosuvastatin [8]. Furthermore, recent cohort studies revealed an increased risk of abnormally high muscle enzymes and an increased hazard ratio for rhabdomyolysis with rosuvastatin in comparison to atorvastatin [9,10]. Moreover, in the PLANET-1 trial, a randomized controlled study comparing the effects of atorvastatin 80mg vs rosuvastatin 40mg in patients with a background of diabetic nephropathy, atorvastatin demonstrated superior renal protective properties compared to rosuvastatin [11]. Additionally, a recent systematic review and meta-analysis reported that administering rosuvastatin at high doses (>20mg) increased the chances of liver function test abnormalities [12]. Despite the documented occurrence of myopathy and renal and liver toxicity associated with rosuvastatin, the drug continues to dominate the global market due to its superior cholesterol-lowering effectiveness compared to other agents in its class.

Various risk factors in statin-induced myopathy are low body mass index, vigorous exercise, diminished hepatic and renal function, advanced age, multiple comorbidities, alcoholism, female sex, major surgery or trauma [13]. Although controversial, vitamin D deficiency is considered a major modifiable risk factor associated with statin-induced myopathy. Results from three distinct study designs (prospective, case series, and cross-sectional) have shown a positive correlation of statin-induced myopathy with vitamin D deficiency [14-16]. On the other hand, the results of two other retrospective reviews have shown no significant relationship between statin-induced myopathy and vitamin D [17,18]. Despite this controversy, all of these reports agree that vitamin D could be considered a therapeutic option only if substantial evidence has been established.

## Conclusions

Statins are widely recognized as a cornerstone of treatment for the prevention of CVD due to their proven efficacy and safety profile. In our case series, the initiation of rosuvastatin, either alone or in conjunction with vitamin D deficiency, was identified as the primary factor linked to the development of myopathy, AKI, and liver injury. Despite the challenges posed by these adverse effects, all patients experienced successful recovery upon discontinuation of rosuvastatin, with significant improvements in muscle strength and the restoration of liver and kidney function observed within weeks. However, it is important to note that these complications resulted in substantial morbidity and healthcare costs. This series adds valuable insights to the ongoing discussions regarding the safety of rosuvastatin and underscores the critical importance of vigilant monitoring and careful consideration when prescribing this medication. To guarantee the safe and efficient use of rosuvastatin in clinical practice, more pharmacovigilance is necessary.
